# Decanoic acid modulates cerebral metabolism and attenuates ischemic injury in a mouse model

**DOI:** 10.1007/s11011-026-01832-w

**Published:** 2026-03-28

**Authors:** Antonio Ítalo Santos Nunes, Lurian Caetano David, Daniel Pereira Cavalcante, Gustavo Almeida de Carvalho, Eduardo Rosa Silva, Jacqueline Alves Leite, Nelson Roberto Antoniosi Filho, Mauro Cunha Xavier Pinto

**Affiliations:** 1 Departamento de Farmacologia, Instituto de Ciências Biológicas, Universidade Federal de Goias, Goiânia, GO Brazil; 2 Instituto de Química, Universidade Federal de Goias, Goiânia, GO Brazil; 3 Departamento de Farmacologia, Laboratório de Neuroquímica e Neurofarmacologia, Universidade Federal de Goiás, Instituto de Ciências Biológicas, Av. Esperança, S/N, UFG, Prédio ICB II, Sala 114, 74690-900 Goiânia-GO, Brazil

**Keywords:** Stroke, Decanoic acid, Neuroprotection, Neuroinflammation, Oxidative stress

## Abstract

Cerebral ischemia, resulting from acute interruption of cerebral blood flow, is a leading cause of long-term cognitive and motor disabilities, and current therapeutic options remain limited. Decanoic acid, a medium-chain fatty acid with antioxidant and anti-inflammatory properties, has been proposed as a potential neuroprotective agent. This study evaluated the neuroprotective and metabolic effects of decanoic acid in a murine model of permanent middle cerebral artery occlusion (MCAO). Male mice received decanoic acid (62.5, 125, or 250 mg/kg, oral gavage) or vehicle for 7 days after MCAO. Infarct volume was quantified by TTC staining. Motor and cognitive performance were assessed using the Cylinder, Limb Clasping, Object Location, and Novel Object Recognition tests. Metabolomic profiling (HS/GC–MS) of cortical tissue was performed to characterize ischemia-induced metabolic disturbances and treatment-related modulation of lipid and energy metabolism. Oxidative stress markers, antioxidant enzyme activities, and inflammatory cytokines (ELISA) were also measured in brain tissue. Decanoic acid significantly reduced total infarct volume and improved motor coordination and memory performance. Metabolomic analysis revealed that DA modulated metabolites disrupted by ischemia, partially restoring lipid and aldehyde homeostasis. Furthermore, treatment decreased lipid peroxidation, enhanced antioxidant enzyme activities, and effectively reversed the ischemia-induced elevation of pro-inflammatory cytokines, thereby neutralizing the neuroinflammatory response. These findings suggest that decanoic acid may exert a coordinated regulatory effect, contributing to neuroprotection through the modulation of cerebral metabolic homeostasis and attenuation of oxidative and inflammatory stress, and supporting its potential as a therapeutic candidate for ischemic brain injury.

**Graphical Abstract:**

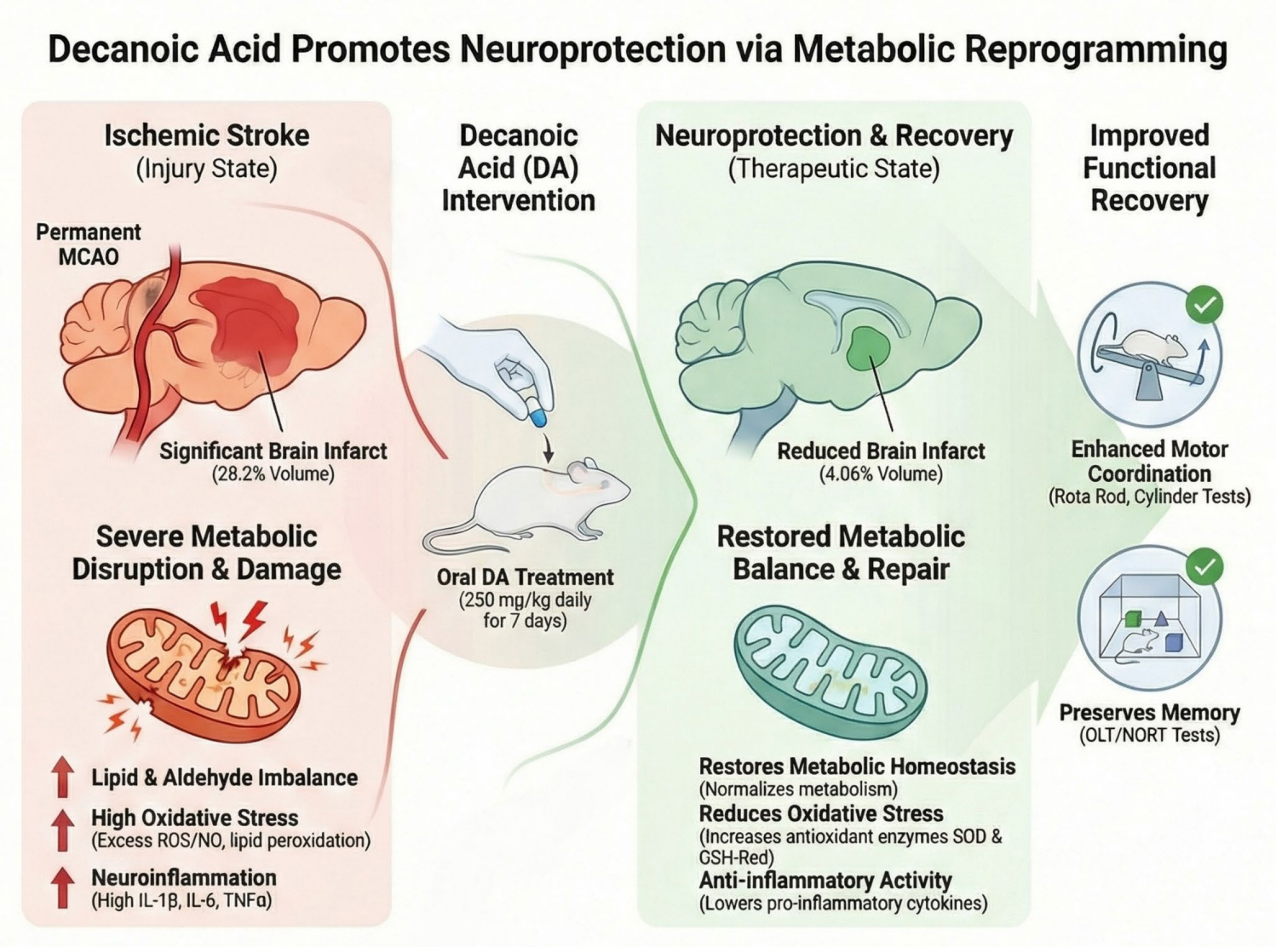

**Supplementary Information:**

The online version contains supplementary material available at 10.1007/s11011-026-01832-w.

## Introduction

Cerebral ischemia is defined as a reduction of either focal or global cerebral blood flow, leading to a critical decrease in the delivery of oxygen and glucose to the central nervous system (CNS). This metabolic insufficiency disrupts cellular homeostasis and ultimately results in neuronal death (Edwards et al. 2017; Zhao et al. 2022). In response to the energy deficit, cells within the ischemic territory shift to anaerobic glycolysis; however, the adenosine triphosphate (ATP) generated through this pathway is insufficient to sustain neuronal function. The severity and duration of the ischemic insult determine the extent of tissue injury (Brouns and De Deyn 2009; Xu et al. 2020; Zhao et al. 2022). Oxygen and glucose deprivation triggers a multifactorial pathophysiological cascade that culminates in anatomical tissue loss and subsequent neurological deficits. This cascade involves profound alterations in energy metabolism, disruption of ion gradients, calcium overload, excitotoxicity, oxidative stress, activation of neuroinflammatory processes, and the induction of cell death pathways, including apoptosis and autophagy, all of which contribute to the progression of ischemic pathology (Cavalcante et al. 2025; Marques 2022; Pinto et al. 2012, 2014; Szydlowska and Tymianski 2010).

In our previous study, DESI–MS imaging revealed an increase in decanoic acid levels in the hippocampus after 30 days of chronic cerebral hypoperfusion, suggesting that this lipid may be involved in the biochemical response to ischemia and that the decanoyl pathway participates in the pathophysiology of chronic ischemia, representing a potential target for pharmacological intervention (Severiano et al. 2020). Decanoic acid, also known as capric acid, is a medium-chain saturated fatty acid consisting of ten carbon atoms. It is one of the principal components of the medium-chain triglyceride (MCT) ketogenic diet and occurs naturally in various dietary sources, including coconut oil and palm kernel oil (Chang et al. 2016; Damiano et al. 2020).

The neuroprotective potential of decanoic acid has been initially supported by evidence derived from target-based and cellular studies. Mechanistic in vitro investigations have identified decanoic acid as a non-competitive antagonist of AMPA-type ionotropic glutamate receptors, a property that may contribute to the reduction of excessive excitatory neurotransmission (Augustin et al. 2018; Chang et al. 2016). This apparent anti-excitotoxic mechanism is complemented by findings in SH-SY5Y neuroblastoma cells, in which decanoic acid modulated peroxisomal metabolism by increasing acyl-CoA oxidase 1 activity and promoting peroxisomal proliferation, thereby suggesting a role in the regulation of cellular lipid homeostasis (Baldwin et al. 2024). Collectively, these molecular and cellular effects are consistent with the neuroprotective activity reported for decanoic acid in multiple experimental models.

In models of chronic neurodegenerative disorders, decanoic acid has exhibited a broad spectrum of biological effects across distinct experimental systems. In a *Caenorhabditis elegans* model of Parkinson’s disease, decanoic acid isolated from *Holothuria leucospilota* attenuated dopaminergic neurodegeneration and oxidative stress, an effect associated with activation of the insulin/insulin-like growth factor signaling (IIS)/DAF-16 pathway (Sanguanphun et al. 2022). Similarly, in 5xFAD mouse models of Alzheimer’s disease, decanoic acid treatment restored AMPA receptor-mediated calcium signaling in hippocampal astrocytes and neurons, suggesting a potential mechanism for the normalization of glutamatergic neurotransmission under conditions of advanced neurodegeneration (Abghari et al. 2023).

Within the context of acute brain injury, the therapeutic potential of decanoic acid in experimental cerebral ischemia has received increasing attention. Sharma et al. (2023a) reported that oral administration of decanoic acid (120 mg/kg) conferred protection against ischemia–reperfusion injury in rats subjected to middle cerebral artery occlusion (MCAO). Both prophylactic and post-insult treatment paradigms significantly reduced infarct volume and neurological deficits, while also attenuating inflammatory responses, oxidative stress, and apoptotic signaling, in parallel with increased expression of neurotrophic factors, including BDNF and TrkB. Notably, the reported reduction in the AMPA receptor GluR1 subunit further supports a potential mechanism involving modulation of excitotoxic signaling (Sharma et al. 2023a).

Although the available evidence collectively supports the therapeutic relevance of decanoic acid, the mechanisms by which it may influence cortical metabolic reprogramming in models of permanent focal ischemia remain insufficiently characterized. Addressing this gap, the present study investigated the effects of a 7-day oral treatment with decanoic acid in a rodent model of cerebral ischemia induced by permanent middle cerebral artery occlusion (MCAO). By integrating histological, behavioral, biochemical, and metabolomic assessments, this approach provides an experimental framework for evaluating mechanisms potentially associated with attenuation of ischemic injury and promotion of neuroprotection.

## Materials and methods

### Animals

Adult male Swiss mice (40–50 g; 8–10 weeks of age) were employed in the experiments. Upon arrival, animals were randomly allocated into five experimental groups (*n* = 5–10 per group, depending on the procedure and sample size calculations). Mice were housed under controlled temperature conditions and maintained on a 12-h light/dark cycle, with unrestricted access to standard chow and water. All animals were obtained from the animal facility of the Federal University of Goiás. The study protocol was reviewed and approved by the Institutional Animal Care and Use Committee at UFG (CEUA Protocol 48/2023).

### Drug preparation and treatment of mice

**Fig. 1:**
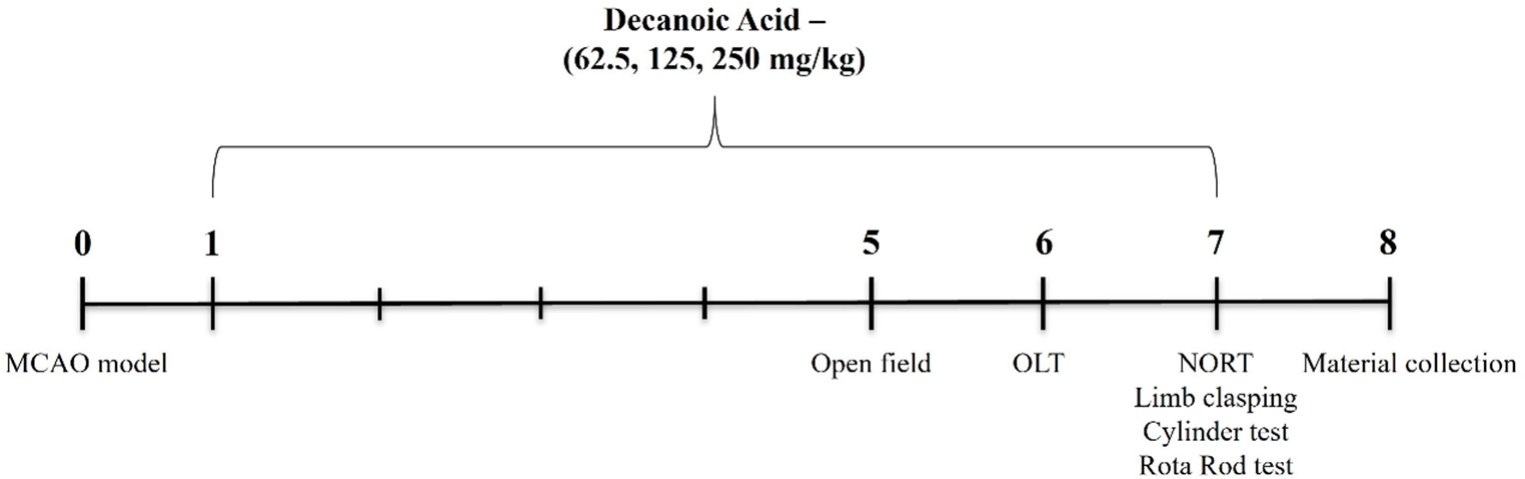
**Schematic representation of experimental design.** On day 0, permanent middle cerebral artery occlusion (MCAO) surgery was performed to induce focal cerebral ischemia. On day 1, daily oral (p.o.) treatment with decanoic acid at doses of 62.5, 125, or 250 mg/kg, or with vehicle (0.5% methylcellulose), was initiated and continued for seven consecutive days. Behavioral assessments were conducted on day 7 to evaluate motor and cognitive performance. On day 8, animals were euthanized according to the established protocol, and brain tissues were collected for subsequent histological, biochemical, and metabolomic analyses. This schematic outlines the temporal organization of the surgical, therapeutic, and analytical phases of the study

Decanoic acid (Sigma-Aldrich Cat# D3879) was utilized as a test compound in the experimental protocol. For administration, the substance was prepared as a suspension in 0.5% methylcellulose (Sigma-Aldrich, Cat# M0512) and delivered orally (p.o.) by gastric gavage at a standardized volume of 10 mL/kg. Focal cerebral ischemia was produced through permanent middle cerebral artery occlusion (MCAO), following established procedures for induction of ischemic injury (Sect.  2.3). Treatment was initiated 24 h after surgery (on Day 1) and continued once daily for seven consecutive days with decanoic acid at doses of 62.5, 125, or 250 mg/kg, or with a vehicle (0.5% methylcellulose) and delivered orally by gastric gavage at a standardized volume of 10 mL/kg. (Fig. 1). The mice were then randomly assigned to five predefined experimental groups: (1) Sham group, consisting of animals that underwent identical surgical manipulation without actual occlusion of the artery; (2) Vehicle group, comprising mice subjected to MCAO and treated with 0.5% methylcellulose; (3) 62.5 mg/kg group, in which ischemic animals received 62.5 mg/kg of decanoic acid; (4) 125 mg/kg group, treated with 125 mg/kg of decanoic acid after MCAO; and (5) 250 mg/kg group, treated with 250 mg/kg of decanoic acid post-occlusion. This experimental design was established to evaluate the neuroprotective effects of decanoic acid at different doses in cerebral ischemia, with the sham and vehicle groups serving as methodological and pharmacological controls, respectively.

### Permanent Middle Cerebral Artery Occlusion (MCAO) model

Common carotid artery occlusion combined with permanent distal middle cerebral artery occlusion was performed following methodologies previously validated for murine cerebral ischemia (Wayman et al. 2016). All procedures were conducted under inhalational anesthesia with 5% isoflurane. A cervical incision was created using a scalpel to expose the common carotid artery (CCA), which was carefully separated from the vagus nerve using fine forceps and subsequently ligated with a silk suture. After completion of the CCA ligation, the midline neck incision was closed, and each animal was repositioned to allow access to the ipsilateral cranial region. A second incision was made between the retro-orbital sinus and the external acoustic meatus, permitting visualization of the skull and the course of the middle cerebral artery (MCA). Craniotomy was then performed in the exposed area using a dental drill to provide direct access to the distal segment of the MCA. Permanent occlusion of the vessel was achieved by applying electrocauterization (Cautermax^®^), after which the cranial incision was sutured. Following the surgical intervention, mice were allowed to recover from anesthesia under controlled ambient temperatures (25–27 °C). Animals assigned to the sham group underwent exposure of the CCA without MCA manipulation, thereby preventing any potential tissue injury associated with craniotomy while maintaining procedural consistency between groups.

### Motor function assessment

#### Cylinder test

The Cylinder Test was employed to assess forelimb asymmetry and motor coordination in mice subjected to the MCAO procedure. This behavioral paradigm evaluates preferential forelimb use during spontaneous exploratory activity (Schallert et al. 2000). For the essay, each mouse was placed inside a transparent acrylic cylinder (20 cm in diameter; 30 cm in height), and its behavior was continuously recorded for a 5-minute session. Spontaneous rearing behavior was quantified by counting the number of independent wall contacts made by the ipsilateral (non-impaired) and contralateral (impaired) forelimbs, as well as simultaneous bilateral contacts.

A valid contact was defined as the placement of the entire palm against the cylinder wall while the animal was in a vertical weight-bearing posture. Lateral ‘stepping’ movements along the wall were also recorded as separate contacts. To ensure data reliability, only sessions in which the animal performed a minimum of 20 contacts were included in the final analysis. Video inspection was performed frame-by-frame using VLC software by an investigator blinded to the experimental groups. Results were expressed as a percentage of ipsilateral (non-impaired) use relative to the total number of contacts.

#### Limb clasping

The Limb Clasping task was utilized to assess functional motor performance in the murine stroke model. As described in previous literature (Miedel et al. 2017), this assay exploits the innate escape reflex elicited when a mouse is lifted by the tail. A failure to fully abduct the hind limbs and extend the toes during suspension reflects impairment of motor function. In this protocol, animals were gently suspended by the tail for a duration of 10 s while their hindlimb and forelimb movements were recorded using a high-definition (HD) camera. After recording, limb clasping scores were assigned according to predefined evaluative criteria on a scale of 0 to 4. (0- No limb clasping. Normal escape extension. 1- One hind limb exhibits incomplete splay and loss of mobility. Toes exhibit normal splay. 2- Both hind limbs exhibit incomplete splay and loss of mobility. Toes exhibit normal splay. 3- Both hind limbs exhibit clasping with curled toes and immobility. 4- Forelimbs and hind limbs exhibit clasping and are crossed, curled toes and immobility).

#### Rota rod test

The rotarod assay was conducted using a rotating rod apparatus with a 3-cm diameter (INSIGHT, São Paulo, Brazil). Prior to testing, each mouse completed a standardized training session on the device, during which the rod was maintained at a constant rotation speed of 10 rpm. Animals were trained until they achieved the performance criterion of remaining on the rotating spindle for at least 10 s. For the experimental evaluation, the rotarod was operated at a fixed speed of 22 rpm, with a maximum trial duration of 120 s. The latency to fall from the apparatus was recorded individually for each mouse. All animals underwent three consecutive trials under identical conditions, and the mean latency value from these trials was used for subsequent statistical analyses (Shiotsuki et al. 2010).

### Memory assessment

#### Open field test

Spontaneous locomotor activity was assessed using the open-field test, following procedures previously described in the literature (Murtishaw et al. 2018). Each animal was placed individually in an open-field arena measuring 45 × 45 × 45 cm for a 6-minute session of unrestricted exploration. All sessions were recorded and subsequently analyzed using the ANY-maze tracking software. Mice were randomly distributed across the different treatment groups, and the apparatus was thoroughly cleaned between trials to eliminate residual odors or cues. Experimental procedures and data analyses were performed by independent investigators who were blinded to group allocation.

#### Object Location Test (OLT)

The Object Location Test (OLT) was employed to evaluate spatial memory performance in mice (Denninger et al. 2018). This paradigm relies on the animal’s intrinsic preference for novelty, thereby eliminating the need for external reinforcement and reducing potential emotional influences on behavior. Initially, mice were habituated to the open-field arena as previously described, after which they were exposed to two identical objects for a 10-minute exploration period (training phase). Following a 20-minute retention interval, one of the objects was displaced to a new location within the arena, and the animals were permitted to explore both objects for an additional 10 min (test phase). Intact spatial memory is inferred when animals spend proportionally more time exploring the relocated object. The recognition index was computed using the following formula:

$$\begin{aligned}&\:\mathrm{Recognition\:Index\:(\%)}=\\&\frac{\mathrm{Time\:spent\:on\:moved\:object}}{\mathrm{Time\:spent\:on\:moved\:object}+\mathrm{Time\:spent\:on\:unmoved\:object}}\times\:100 \end{aligned}$$

#### Novel Object Recognition Test (NORT)

The Novel Object Recognition Test (NORT) was conducted 24 h after completion of the Object Location Test (OLT) training session. In this essay, one of the objects previously encountered by the animals was substituted with a novel item. Following the replacement, mice were allowed a 10-minute period of unrestricted exploration of both the familiar and novel objects. Animals with preserved recognition memory are expected to display a spontaneous preference for the novel object. Whereas spatial memory assessed by the OLT is strongly dependent on hippocampal integrity, recognition memory engages multiple neural circuits, with hippocampal involvement varying across conditions. Nonetheless, the NORT remains a widely utilized behavioral paradigm for evaluating recognition memory in rodents (Miedel et al. 2017). The recognition index was calculated using the following formula:

$$\begin{aligned}&\:\mathrm{Recognition\:Index\:(\%)}=\\&\frac{\mathrm{Time\:spent\:on\:novel\:object}}{\mathrm{Time\:spent\:on\:novel\:object}+\mathrm{Time\:spent\:on\:familiar\:object}}\times\:100 \end{aligned}$$

### Infarct volume analysis

For morphological evaluation, mouse brains were removed and sectioned into consecutive coronal slices, each 2 mm in thickness, resulting in a total of four sections per animal. The slices were then incubated in a 2% solution of 2,3,5-triphenyltetrazolium chloride (TTC; Sigma-Aldrich, Cat# T8877) for staining. Quantification of infarcted volume was performed using ImageJ software. The total infarct volume was quantified by summing the infarcted areas across all sections and expressing it as a percentage of the total cortical volume (infarct volume / total cortical volume × 100) (Zhang and Chen 2012).

### Biochemical analysis

#### Sample preparation

Sample preparation was performed at the experimental endpoint (Day 8), animals were euthanized and brains were immediately excised. The right cerebral cortex corresponding to the ischemic hemisphere (ipsilateral to the occlusion) was carefully dissected under ice-cold conditions to preserve protein integrity. Tissue samples were processed to obtain brain homogenate supernatants for biochemical and inflammatory analyses. Briefly, each sample was supplemented with 500 µL of ice-cold lysis buffer containing 50 mM sodium fluoride (NaF), 1 mM sodium orthovanadate (Na₃VO₄), 1× SigmaFast protease inhibitor cocktail, and 1% Triton X-100. Samples were incubated on ice for 20–30 min and subsequently sonicated until complete homogenization. Homogenates were then centrifuged at 12,000 × g for 30 min at 4 °C, after which the supernatant was collected, aliquoted, and stored at − 20 °C–− 80 °C until analysis. All biochemical and inflammatory measurements were normalized to the total protein concentration of the corresponding supernatant.

For metabolomic analysis, a separate set of freshly dissected ipsilateral cortical tissue fragments (20 mg) was collected independently, transferred to sealed headspace vials, and analyzed by HS–GC/MS, as described in Sect.  2.7.4.

#### Measurement of free radical content and lipid peroxidation

Reactive oxygen species (ROS) levels were quantified using 2’,7’-dichlorofluorescein diacetate (DCF-DA; Sigma-Aldrich, Cat# D6883), a widely used fluorogenic probe (Siqueira et al. 2004). For this assay, 5 µL of each sample were incubated with 195 µL of DCF-DA (125 µM) at 37 °C for 30 min, protected from ambient light. The oxidation of DCF-DA to its fluorescent product, dichlorofluorescein (DCF), was monitored with a fluorescence plate reader (Cary Eclipse, Varian, USA) using excitation and emission wavelengths of 488 and 525 nm, respectively. Fluorescence values were normalized to total protein concentrations and expressed as a percentage relative to the vehicle group (MCAO + vehicle).

Nitric oxide (NO) production was assessed using 2,3-diaminonaphthalene (DAN; Sigma-Aldrich, Cat# D2625), following the fluorescent detection method previously described by (Misko et al. 1993). In brief, 175 µL of 3.2 mM DAN were added to 5 µL of brain homogenate samples. After incubation for 10 min at 25 °C under light-protected conditions, the reaction was terminated by adding 20 µL of 2.8 M NaOH (Sigma-Aldrich, Cat# 221465). The resulting fluorescent adduct was measured with a fluorescence plate reader (Cary Eclipse, Varian, USA) at excitation and emission wavelengths of 360 and 440 nm, respectively. Data were normalized to protein levels and expressed as a percentage of the vehicle group (MCAO + vehicle).

Lipid peroxidation was evaluated by quantifying thiobarbituric acid (Sigma-Aldrich, Cat# T5500) reactive substances (TBARS) in tissue homogenates and expressed as malondialdehyde (MDA) equivalents, measured spectrophotometrically at 535 nm according to the method of Ohkawa et al. (1979) (Ohkawa et al. 1979). Results were reported as the percentage increase in TBARS (nmol MDA/mg protein) relative to vehicle group (MCAO + vehicle).

#### Measurement of antioxidant enzyme activities

Glutathione reductase (GR) activity was quantified following the method described by Carlberg and Mannervik (1975) (Carlberg and Mannervik 1975). In this assay, 5 µL of each sample were combined with 155 µL of 0.10 M potassium phosphate (Neon, Cat# 01374) buffer containing 0.5 mM EDTA (Dinâmica, Cat# 1593), followed by the addition of 20 µL NADPH (Sigma-Aldrich, Cat# 481973) and 20 µL oxidized glutathione (Sigma-Aldrich, Cat# G4376) (pH 7.6). Enzyme activity was determined based on the oxidation of 1 mol of NADPH per minute, using the molar extinction coefficient of NADPH at 340 nm. Values were normalized to protein concentration and expressed as percentages of the vehicle group (MCAO + vehicle).

Superoxide dismutase (SOD) activity was evaluated using the spectrophotometric procedure previously reported by Marklund and Marklund (1974) (Marklund and Marklund 1974). Samples were incubated in a reaction mixture composed of 50 mM potassium phosphate buffer and 50 mM EDTA (pH 7.4). The reaction was initiated by adding 2 mM pyrogallol (Sigma-Aldrich, Cat# P0381), and the rate of pyrogallol autoxidation was monitored at 420 nm for 5 min at 30-second intervals. Subsequently, 3 µL of the sample were introduced, and absorbance readings were recorded again under identical conditions. Enzymatic activity was normalized to protein content and expressed relative to vehicle group (MCAO + vehicle).

Glutathione S-transferase (GST) activity was determined according to the method of Habig et al. (1974) (Habig et al. 1974). In brief, 5 µL of homogenate were mixed with 175 µL of 0.1 M phosphate buffer containing 1.0 mM EDTA (pH 6.5), supplemented with 1.06 mM reduced glutathione (GSH; Sigma-Aldrich, Cat# G4251) and 1.06 mM 1-chloro-2,4-dinitrobenzene (CDNB; Sigma-Aldrich, Cat# 138630). The reaction components were pipetted directly into quartz cuvettes, and absorbance changes at 340 nm were measured spectrophotometrically. Results were normalized to protein concentration and expressed as percentages relative to the vehicle group (MCAO + vehicle).

Catalase (CAT) activity was measured by monitoring the enzymatic decomposition of hydrogen peroxide (H₂O₂), following the protocol described by Shangari and O’Brien (2006) (Shangari and O’Brien 2006). The reaction was initiated by adding 5 µL of sample to a solution containing 7.5 mM H₂O₂ (Neon, Cat# 01984) prepared in 50 mM potassium phosphate buffer (pH 7.0). The decline in H₂O₂ concentration was recorded spectrophotometrically at 240 nm, with absorbance readings obtained every 15 s over a 15-minute period at 25 °C. Data were normalized to protein levels and expressed as percentages of the vehicle group (MCAO + vehicle).

#### Determination of inflammatory biomarkers by ELISA

Quantification of TNF-α, IL-1β, and IL-6 levels was performed using a Sandwich Enzyme-Linked Immunosorbent Assay (ELISA), following the manufacturer’s specifications (R&D Systems., USA). Microtiter plates were precoated with target-specific monoclonal antibodies, subsequently blocked with BSA/PBS solution, and loaded with 50 µL of standards or samples per well. After the incubation period and subsequent wash steps, streptavidin-HRP conjugate was applied, followed by the addition of the chromogenic substrate and incubation under light-protected conditions. Absorbance readings were obtained at 450 nm, and cytokine concentrations were derived from standard curves ranging from 15.6 to 2000 pg/mL. Final values were expressed in pg/mL for TNF-α (R&D Systems, Cat# DY410-05, RRID: not available), IL-1β (R&D Systems, Cat# DY401-05, RRID: not available), and IL-6 (R&D Systems, Cat# DY406-05, RRID: AB_3068344).

#### Metabolomics analysis of cortical tissue by HS/GC–MS

Volatile metabolites present in the mouse cortex were characterized using an untargeted metabolomic workflow (da Cunha et al. 2025). Approximately 20 mg of freshly dissected cortical tissue were transferred into 10 mL HS–GC vials. All vials were hermetically sealed with PTFE-lined magnetic caps, randomized prior to analysis, and subjected to HS/GC–MS profiling. Volatile compounds were extracted using a Shimadzu AOC-5000 headspace autosampler (Shimadzu, Kyoto, Japan) equipped with a 2500 µL gas-tight syringe and a preheating LHS0 CombiPal module (PAL System, Zwingen, Switzerland). Headspace sampling parameters were as follows: incubation at 180 °C for 60 min with agitation at 500 rpm (cycles of 5 s on/2 s off), pre-purge for 5 s, post-purge for 30 s, syringe temperature of 150 °C, injection volume of 2500 µL, and injection flow rate of 100 mL min⁻¹. Chromatographic separation was performed on a Shimadzu GCMS-QP2010 Ultra system coupled to an NST-100 ms capillary column (25 m × 0.25 mm i.d. × 0.3 μm film thickness; polyethylene glycol stationary phase; NST–Nano Separation Technologies, São Paulo, Brazil). High-purity helium (99.999%; White Martins, Goiânia, Brazil) served as the carrier gas at a constant flow of 1.36 mL min⁻¹ (linear velocity 45.8 cm s⁻¹). The oven temperature program consisted of an initial hold at 30 °C for 5 min, followed by increases to 45 °C and 50 °C (2 °C min⁻¹; 5 min each), a ramp to 120 °C (2 °C min⁻¹), a subsequent increase to 200 °C (6 °C min⁻¹), and a final gradient to 250 °C (5 °C min⁻¹; 10 min), resulting in a total chromatographic run time of 98.33 min. Mass spectrometric detection was carried out using a Shimadzu QP2010 Ultra mass spectrometer (Shimadzu, Kyoto, Japan) operating under electron ionization (EI) at 70 eV. Instrumental conditions included an ion source temperature of 280 °C, a scan speed of 1666 u s⁻¹, and a scan interval of 0.3 s. Tentative metabolite identifications were obtained by matching mass spectra against the NIST17 reference library (National Institute of Standards and Technology, Gaithersburg, MD, USA).

#### Data processing and statistical analysis

Raw HS/GC–MS chromatographic data were processed using LabSolutions GCMSsolution software v.4.50 SP1 (Shimadzu, Kyoto, Japan). Detected peaks were classified according to signal characteristics: peaks with an area greater than zero and an area-to-height (A/H) ratio exceeding 3 were assigned a value of “1,” whereas peaks with zero area or an A/H ratio below 3 were assigned a value of “0.” These binary outputs were compiled into matrices for metabolite frequency assessment, after which the 400 most prevalent compounds were selected for downstream statistical processing (see Supplementary Information). The resulting data matrix (18 samples × 400 variables) was analyzed using MetaboAnalyst 6.0 (www.metaboanalyst.ca) and R version 4.3.2. Data were uploaded under the “Concentrations” format with “Samples in columns” and “Unpaired” selected as the study design. Integrity verification confirmed that all variables were numeric, without missing values, and that the dataset comprised three experimental groups (Sham, MCAO, and DA250). Variables with zero variance were removed automatically. No normalization or data transformation procedures were applied; instead, auto-scaling (mean-centering followed by division by the standard deviation) was used. Exploratory multivariate analysis included the generation of a heatmap using Euclidean distance and Ward’s clustering algorithm, focusing on the top 50 discriminatory features identified through ANOVA. Group separation was further evaluated using Partial Least Squares Discriminant Analysis (PLS-DA). The PLS-DA score plot demonstrated clear separation among the three experimental groups, and metabolites with VIP values > 2.0 were considered key contributors to class discrimination.

### Statistical analyses

Sample size calculations were conducted using G*Power software (version 3.1.9.2), with an α level set at 0.05 and statistical power fixed at 0.8. Effect sizes of 0.4 for behavioral analyses and 0.25 for biochemical measurements were incorporated into the estimations. Data are presented either as mean ± standard error of the mean (SEM) or as median with interquartile range (IQR), depending on the underlying distribution. Parametric datasets were analyzed using one-way ANOVA followed by Dunnett’s post hoc test for multiple comparisons, whereas nonparametric datasets were evaluated using the Kruskal–Wallis test followed by Dunn’s post hoc procedure. Statistical significance was defined as *p* < 0.05.

## Results

### Decanoic acid promotes a decrease in ​​ischemia and motor deficits

**Fig. 2:**
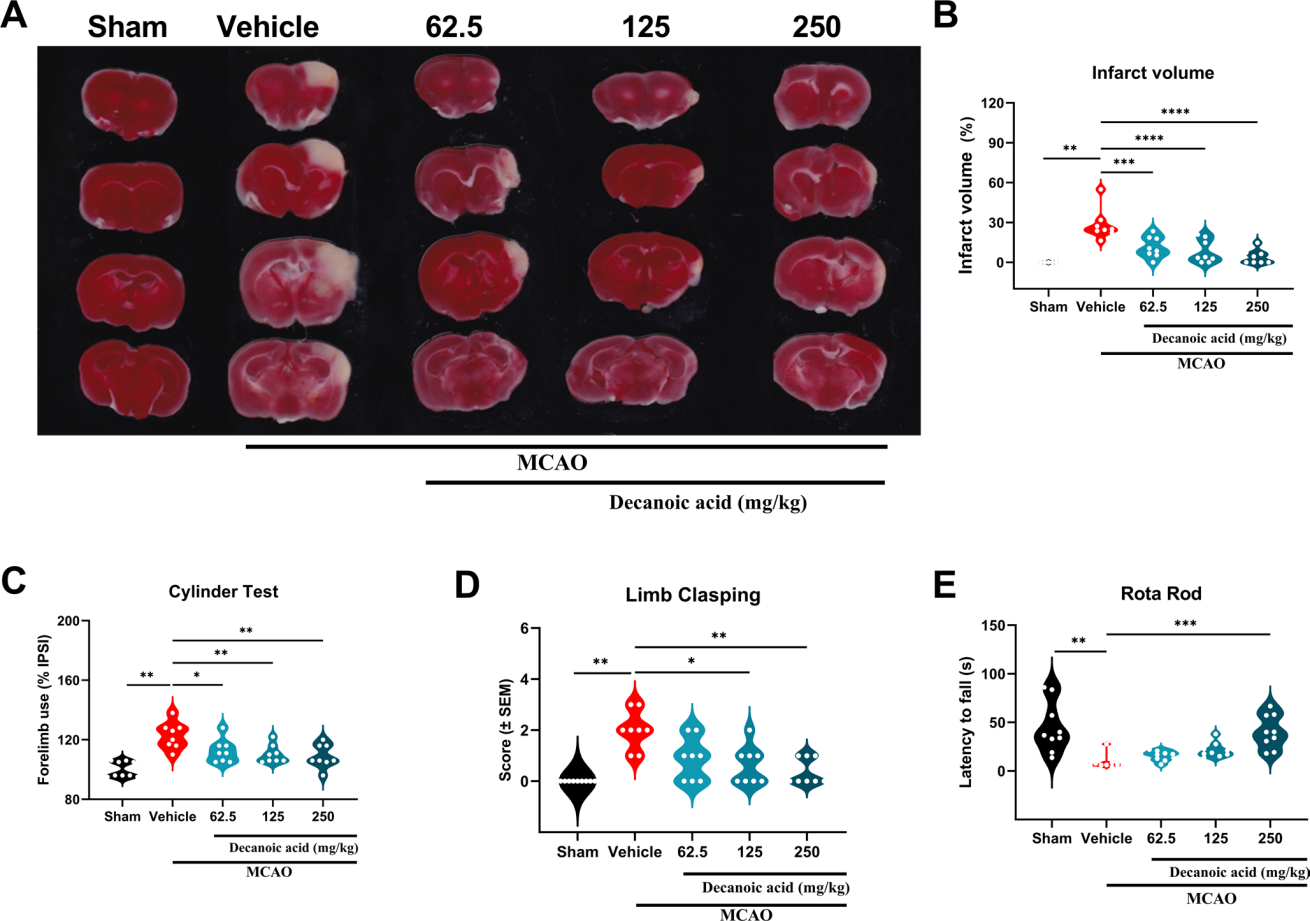
Decanoic acid reduces infarct area and improves ischemia-induced motor deficits. (**A**) Representative TTC-stained brain sections showing the extent of ischemic injury across groups. (**B**) Quantification of total infarct volume based on densitometric analysis of non-viable tissue across all coronal sections, demonstrating a significant reduction in cumulative lesion size following treatment (**C**) Percentage of forelimb use in the cylinder test, reflecting post-ischemic asymmetry in motor exploration. (**D**) Neurological deficit score in the limb-clasping test, assessing motor impairment and postural abnormalities after cerebral injury. (**E**) Latency to fall in the rotarod test, indicating coordination and motor balance. Data are expressed as mean ± SEM and analyzed using one-way ANOVA followed by Dunnett’s post hoc test for parametric data, panels **B**, **C**, and **E**. Data in panel D are expressed as median and interquartile range (IQR) and were analyzed by the Kruskal–Wallis test followed by Dunn’s post hoc test (nonparametric data). p < 0.05. n = 8–9 per group.

The neurorepair potential of decanoic acid was first evaluated in an experimental model of cerebral ischemia. Following a 7-day treatment regimen, decanoic acid administration resulted in a significant reduction in ischemic lesion size. A statistically significant difference was seen between the Sham group and the Ischemia group (28.2 ± 4.12%; *P* < 0.05), confirming the validity of the MCAO model. Treatment with decanoic acid at doses of 62.5 mg/kg (11.37 ± 2.78%; *P* < 0.05), 125 mg/kg (8.12 ± 3.08%; *P* < 0.05), and 250 mg/kg (4.06 ± 1.85%; *P* < 0.05) significantly reduced infarct size compared with the ischemia group (MCAO) (Fig. 2A–B). These results show that all tested doses effectively mitigated ischemic injury.

In parallel, decanoic acid treatment attenuated ischemia-induced motor deficits, Fig. 2E as shown by improved performance in the limb-clasping, cylinder, and rotarod tests. In the limb-clasping test, treatment with 125 mg/kg (score: 0.5, IQR: 0–2; *P* < 0.05) and 250 mg/kg (score: 0, IQR: 0–1; *P* < 0.05) significantly reduced neurological deficit scores relative to the Ischemia group (Fig. 2C). In the cylinder test, mice treated with 62.5 mg/kg (112.30 ± 2.75%; *P* < 0.05), 125 mg/kg (110.30 ± 2.12%; *P* < 0.05), and 250 mg/kg (108.90 ± 2.77%; *P* < 0.05) exhibited greater forelimb use symmetry compared with the Ischemia group (Fig. 2D). Likewise, in the rotarod test, the 250 mg/kg dose (40.48 ± 5.72; *P* < 0.05) significantly increased latency to fall, indicating improved motor coordination. Collectively, these findings demonstrate that decanoic acid reduces infarct size and improves motor deficits.

### Decanoic acid promotes an improvement in spatial and contextual memory

**Fig. 3:**
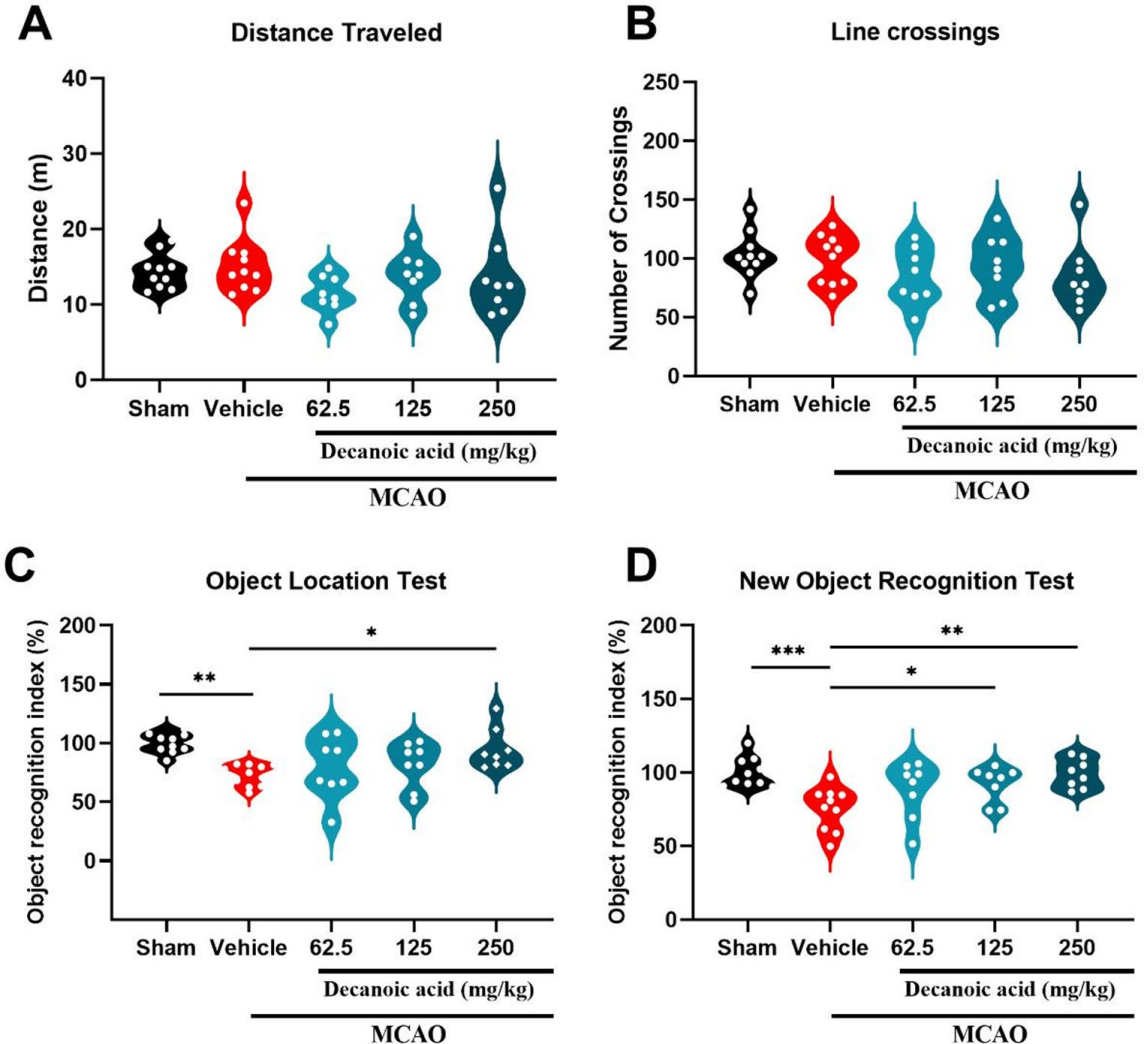
Decanoic acid improves spatial and recognition memory without altering spontaneous locomotion. (**A**–**B**) Open-field test showing that locomotor activity remained unchanged after treatment, confirming that subsequent cognitive improvements are not attributable to motor alterations. (**C**) Recognition index in the Object Location Test (OLT), reflecting the ability to discriminate the relocated object. (**D**) Recognition index in the Novel Object Recognition Test (NORT), assessing recognition memory and preference for novelty. Data is presented as mean ± SEM and analyzed by one-way ANOVA followed by Dunnett’s post hoc test. *p* < 0.05; *n* = 8–10 per group

To assess spatial and recognition memory, the Object Location Test (OLT) and Novel Object Recognition Test (NORT) were performed. Prior to these evaluations, an open-field test was conducted to measure spontaneous locomotor activity. The results showed that the 7-day treatment with decanoic acid after the MCAO procedure did not affect spontaneous locomotion (Fig. 3A–B) when compared with the negative (Sham) or positive (MCAO) control groups. In the OLT, treatment with 250 mg/kg decanoic acid (94.56 ± 6.17%; *P* < 0.05) significantly increased the percentage of investigation directed toward the displaced object compared with the ischemic group (MCAO) (Fig. 3C), indicating improved spatial memory. Similarly, in the NORT, administration of 125 mg/kg (92.31 ± 4.15%; *P* < 0.05) and 250 mg/kg (99.35 ± 3.49%; *P* < 0.05) significantly increased exploration of the novel object relative to the ischemic group (Fig. 3D), demonstrating preserved recognition memory. These findings indicate that decanoic acid maintains both spatial and recognition memory in a rodent model of focal cerebral ischemia, further supporting its potential as a neuroprotective compound.

### Metabolomic profiling of decanoic acid treatment in the MCAO model

Once neuroprotection was seen following decanoic acid treatment in the MCAO model, metabolomic analysis was conducted to characterize ischemia-induced metabolic disturbances and to assess whether decanoic acid (250 mg/kg) exerts its effects by modulating lipid and energy metabolism. The PLS-DA analysis revealed a clear separation among the Sham, MCAO, and DA_250 groups across the first two components (Component 1: 9.5%; Component 2: 8.0%). Sham animals clustered consistently in a distinct region of the plot, while the MCAO group formed a separate cluster, confirming the metabolic alterations induced by ischemia. Treatment with decanoic acid (250 mg/kg) resulted in a distinct metabolic profile, clearly separated from the MCAO group, and positioned closer to the Sham group, suggesting a partial recovery of metabolic homeostasis.

**Fig. 4:**
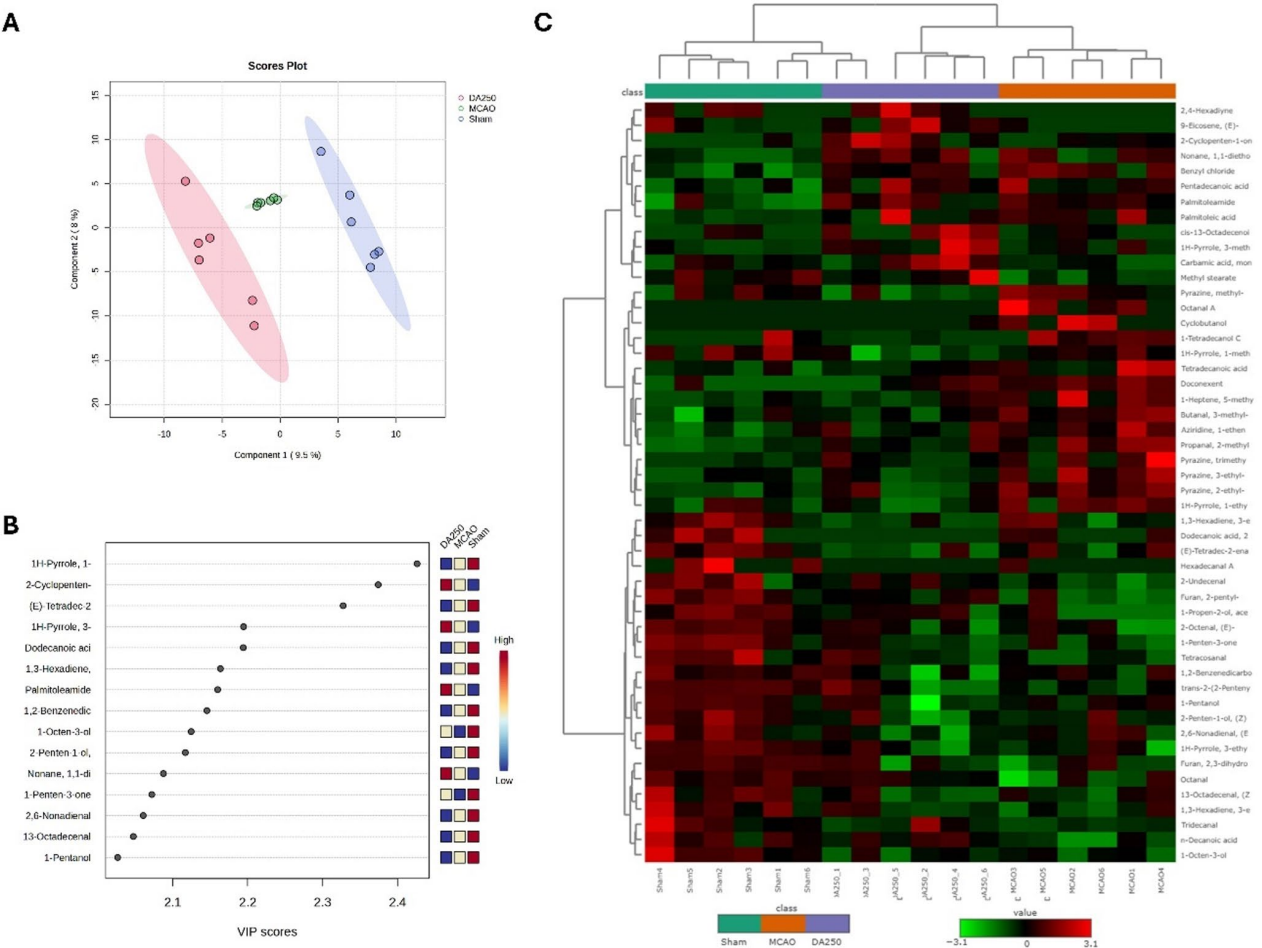
Metabolomic profiling of the Sham, MCAO, and decanoic acid–treated (DA_250) groups. (**A**) PLS-DA score plot showing clear separation among groups across the first two components, indicating metabolic alterations induced by ischemia and partial restoration by decanoic acid. (**B**) VIP scores identifying the main discriminant metabolites, including compounds associated with oxidative stress, lipid metabolism, and reactive aldehydes. (**C**) Heatmap of the top 50 discriminant metabolites, highlighting distinct abundance patterns among groups and demonstrating that decanoic acid modulates lipids, heterocyclic compounds, aldehydes, and alcohols toward a profile closer to Sham. *n* = 6 per group

The VIP score analysis (> 2.0) identified the metabolites contributing most to this discrimination, including 1-methyl-1 H-pyrrole, 2-Pentyl-2-cyclopenten-1-one, tetradec-2-ene, 3-methyl-1 H-pyrrole, dodecanoic acid, 3-ethyl-1,3-hexadiene, and palmitoleamide, as well as several aldehydes and alkanols such as 1-pentanol, 13-octadecanal, and 2,6-nonadienal, all associated with oxidative stress and lipid remodeling (Fig. 4). The heatmap showed that metabolites such as palmitoleamide and hexadecanal were elevated in the MCAO group, whereas compounds like 1-methyl-1 H-pyrrole-1, and 3-ethyl-1,3-hexadiene were modulated by decanoic acid, approaching the metabolic profile of the Sham group. These findings suggest that treatment partially reversed ischemia-induced metabolic disturbances, highlighting the potential of decanoic acid to restore cerebral metabolic balance.

The hierarchical clustering heatmap was generated using normalized and autoscaled data, with the Euclidean distance measure and Ward clustering method (Fig. 4). The top 50 discriminant metabolites were selected based on statistical significance (T-test/ANOVA), and abundance values were displayed using a red/green color scale (red = higher abundance; green = lower abundance). The overview mode was applied for visualization, and a group annotation legend was included to highlight sample classification (Sham, MCAO, and DA_250). The analysis revealed a clear segregation of the three experimental groups. Sham animals clustered together and exhibited a metabolic profile distinct from MCAO animals, which showed pronounced alterations consistent with ischemia-induced metabolic disruption. Treatment with decanoic acid (250 mg/kg) produced an intermediate profile, clustering separately from MCAO and partially overlapping with Sham, suggesting a restorative effect of the treatment on ischemia-associated metabolic imbalances. Several lipid-related metabolites, such as palmitoleamide, hexadecanal, and tetradecanoic acid, were upregulated in the MCAO group, while compounds including 1-methyl-1 H-pyrrole, 2-Pentyl-2-cyclopenten-1-one, and 2-penten-1-ol showed higher relative abundance in DA_250-treated animals. These patterns indicate that decanoic acid modulates lipid, aldehyde, and heterocyclic compound metabolism, shifting the metabolic signature toward that observed in Sham animals and reinforcing its potential to normalize cerebral metabolism after ischemic injury,

**Fig. 5:**
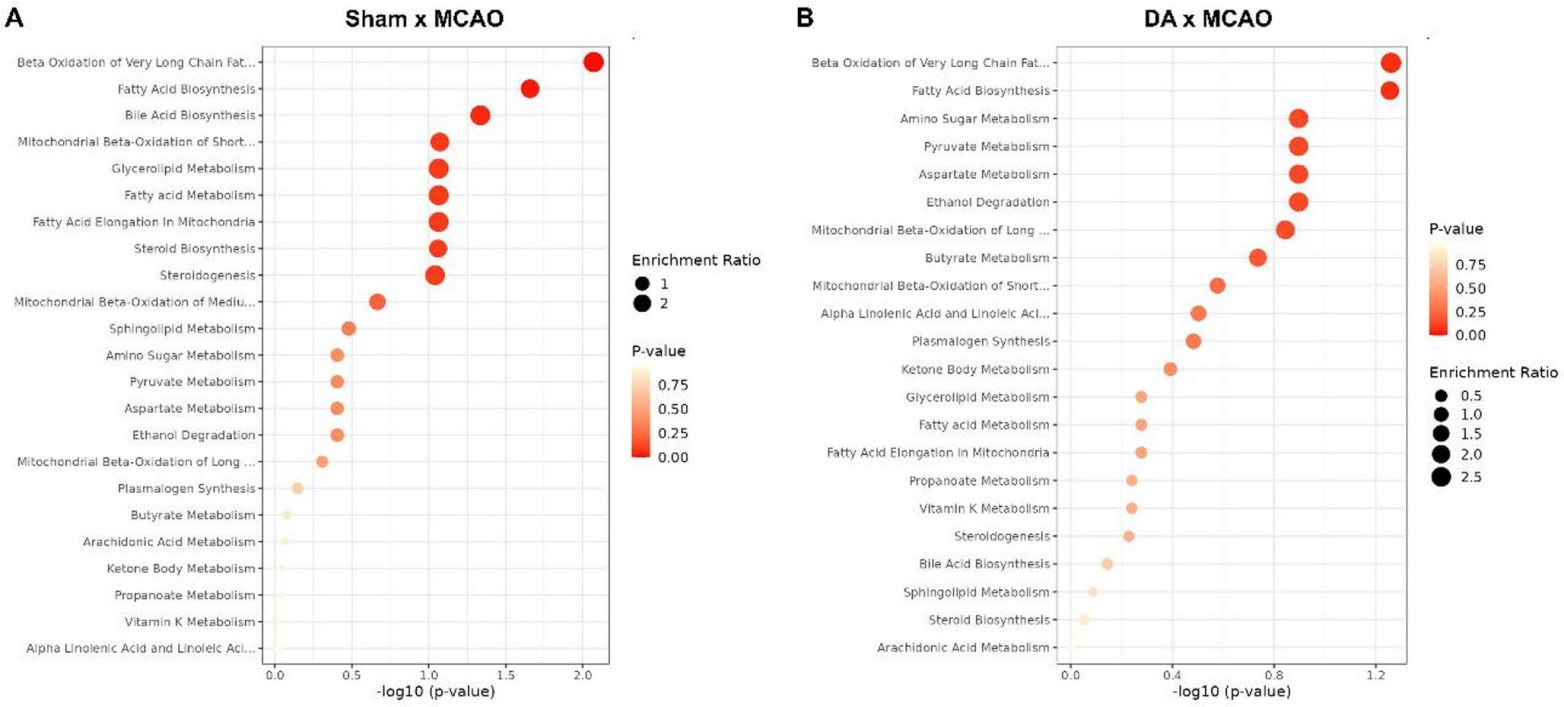
Pathway enrichment analysis of metabolomic alterations. (**A**) Comparison between Sham and MCAO groups revealed significant enrichment of pathways related to polyunsaturated fatty acid (PUFA) metabolism, mitochondrial and peroxisomal β-oxidation, fatty acid biosynthesis, sphingolipid metabolism, and bile acid biosynthesis, indicating profound lipid metabolic disturbances caused by ischemia. (**B**) Comparison between MCAO and MCAO + DA_250 groups demonstrated partial restoration of mitochondrial fatty acid turnover, with additional modulation of sulfite/sulfate metabolism, butyrate metabolism, amino sugar metabolism, and aspartate metabolism. n = 6 per group.

Pathway enrichment analysis comparing the control group (Sham) and the ischemic group (MCAO) revealed marked alterations in metabolic pathways, with the metabolite sets related to lipid metabolism (Fig. 5). The most significant associations were observed for *beta-oxidation of very long chain fatty acids*, *fatty acid biosynthesis*, and *bile acid biosynthesis*. Additional enriched pathways included *mitochondrial beta-oxidation of short-chain fatty acids*, *glycerolipid metabolism*, and *fatty acid elongation in mitochondria*. Other relevant pathways such as *steroid biosynthesis*,* steroidogenesis*, and *sphingolipid metabolism* were also detected, although their statistical support decreased after multiple testing correction. Collectively, these results indicate a prominent disruption of lipid metabolic processes, particularly those associated with fatty acid catabolism and mitochondrial β-oxidation, which are intricately linked to oxidative stress, membrane remodeling, and bioenergetic imbalance during cerebral ischemia.

To further investigate the impact of decanoic acid treatment after ischemia, pathway enrichment analysis was performed comparing the group MCAO + Decanoic Acid (250 mg/kg) and MCAO groups (Fig. 5B). The most enriched pathways included *beta-oxidation of very long chain fatty acids* and *fatty acid biosynthesis*, indicating partial recovery of mitochondrial fatty acid turnover. Moderate enrichment was also observed for *amino sugar metabolism*, *pyruvate metabolism*, and *aspartate metabolism*, suggesting improved oxidative energy coupling. Other pathways, such as *butyrate metabolism*,* alpha-linolenic and linoleic acid metabolism*, and *plasmalogen synthesis*, were also enriched, reflecting enhanced lipid remodeling and mitochondrial adaptability. Taken together, these findings indicate that decanoic acid treatment partially restored mitochondrial β-oxidation and lipid biosynthetic activity while promoting recovery of amino acid–related metabolic pathways. This metabolic reorganization suggests improved redox homeostasis and energy efficiency, reinforcing the neuroprotective potential of decanoic acid after cerebral ischemia.

**Table 1:** Differentially regulated metabolites in SHAM, MCAO, and MCAO + DA groups

Metabolite	Sham (A)	MCAO (B)	DA (C)	*p*-value	B/A	B/C
2-methyl-propanal	1.202 ± 0.955	5.539 ± 2.386	3.494 ± 1.557	0.002	4.609	1.585
2-pentyl-furan	0.644 ± 0.040	0.557 ± 0.041	0.604 ± 0.024	0.003	0.865	0.922
1-Penten-3-one	0.412 ± 0.031	0.356 ± 0.028	0.362 ± 0.023	0.007	0.865	0.983
3-methyl-butanal	5.376 ± 2.467	9.312 ± 1.777	6.076 ± 1.508	0.008	1.732	1.533
n-Decanoic acid	0.019 ± 0.008	0.007 ± 0.006	0.016 ± 0.004	0.008	0.349	0.422
Acetate 1-Propen-2-ol	3.680 ± 1.068	0.975 ± 1.574	2.287 ± 1.276	0.01	0.265	0.426
1-Octen-3-ol	0.827 ± 0.176	0.575 ± 0.118	0.576 ± 0.131	0.011	0.694	0.998
1-ethenyl-aziridine	0.058 ± 0.057	0.193 ± 0.072	0.131 ± 0.073	0.012	3.356	1.473
Octanal	0.318 ± 0.038	0.172 ± 0.120	0.276 ± 0.034	0.012	0.54	0.622
2-(2-Pentenyl)furan	0.196 ± 0.009	0.136 ± 0.023	0.138 ± 0.061	0.026	0.696	0.989
3-ethyl-2-methyl-1,3-Hexadiene	0.160 ± 0.020	0.135 ± 0.015	0.141 ± 0.013	0.043	0.845	0.962
Methyl stearate	0.239 ± 0.022	0.201 ± 0.018	0.241 ± 0.041	0.047	0.84	0.832
Tridecanal	0.230 ± 0.074	0.141 ± 0.013	0.178 ± 0.065	0.049	0.612	0.791

Data are presented as mean ± SD of relative abundance (peak area); p-values from one-way ANOVA and fold changes between groups. All analyses were performed using a standardized mass of 20 mg of cortical tissue per sample.

The comparative analysis identified metabolites significantly modulated among groups, with mean values expressed as relative abundance (peak area) in arbitrary units (AU), as confirmed by ANOVA (*p* < 0.05) and Dunnett’s post-hoc tests (Table 1). In the MCAO group, several aldehydes derived from lipid peroxidation were reduced, including octanal (0.318 ± 0.038 vs. 0.172 ± 0.120; *p* = 0.008) and tridecanal (0.230 ± 0.074 vs. 0.141 ± 0.013; *p* = 0.030), both reflecting membrane oxidative degradation. Treatment with decanoic acid partially restored these levels (octanal: 0.276 ± 0.034, *p* = 0.054; tridecanal: 0.178 ± 0.065, *p* = 0.441). Similarly, 2-pentyl-furan, a recognized biomarker of linoleic acid oxidation, was reduced in ischemic animals (0.644 ± 0.040 vs. 0.557 ± 0.041; *p* = 0.002) and showed a recovery trend after treatment (0.604 ± 0.024; *p* = 0.073). Other lipid oxidation products, including 1-penten-3-one and 2-(2-pentenyl) furan, also decreased under ischemia (*p* < 0.05) but remained unchanged following treatment (*p* > 0.60).

In parallel, metabolites associated with energy metabolism and oxidative stress were elevated in the MCAO group. 2-methyl-propanal, a by-product of valine catabolism, increased markedly (1.202 ± 0.955 vs. 5.539 ± 2.386; *p* = 0.002), with partial but non-significant reduction in treated animals (3.494 ± 1.557; *p* = 0.104). 3-methyl-butanal, linked to leucine metabolism, was also elevated (5.376 ± 2.467 vs. 9.312 ± 1.777; *p* = 0.008) and attenuated by treatment (6.076 ± 1.508; *p* = 0.022). 1-ethenyl-aziridine, a reactive heterocyclic compound, exhibited a strong increase (0.058 ± 0.057 vs. 0.193 ± 0.072; *p* = 0.006), followed by partial normalization (0.131 ± 0.073; *p* = 0.226). This confirms efficient systemic uptake and metabolic incorporation of decanoic acid during ischemia.

Importantly, levels of n-decanoic acid, the compound administered at 250 mg/kg, were significantly reduced in ischemic animals (0.019 ± 0.008 vs. 0.007 ± 0.006; Sham vs. MCAO, *p* = 0.006; fold = 0.349), but were markedly restored following treatment (0.016 ± 0.004; *p* = 0.037; fold = 0.422). This recovery confirms the effective systemic delivery and cerebral bioavailability of decanoic acid under ischemic conditions, demonstrating that the compound not only reaches brain tissue but also modulates lipid homeostasis and mitochondrial β-oxidation. Together, these findings support the dual role of decanoic acid as both a therapeutic neuroprotective agent and a metabolic modulator capable of reestablishing energetic and lipid balance after ischemic injury.

### Decanoic acid promoted a decrease in reactive oxygen species

**Fig. 6:**
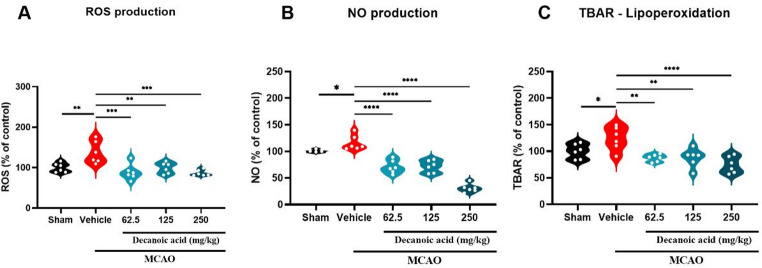
Decanoic acid reduces the production of reactive oxygen species (ROS). (**A**) Levels of reactive oxygen species (ROS) in brain tissue, significantly reduced in all treated groups. (**B**) Nitric oxide (NO) concentrations, a key marker of nitrosative stress, also decreased after treatment. (**C**) Lipid peroxidation levels assessed by thiobarbituric acid reactive substances (TBARS), showing a reduction in membrane oxidative damage in comparison with the MCAO group. Data are presented as mean ± SEM and analyzed using one-way ANOVA followed by Dunnett’s post hoc test. p < 0.05; n = 6 per group.

After confirming the neuroprotective effects of decanoic acid in the MCAO model, metabolomic profiling revealed alterations consistent with modulation of lipid and energy metabolism, prompting further analyses of oxidative stress parameters. Decanoic acid treatment significantly attenuated the production of reactive oxygen species (ROS) in mice subjected to cerebral ischemia. ROS levels were markedly reduced in the groups treated with 62.5 mg/kg (88.59 ± 7.91%; *P* < 0.05), 125 mg/kg (99.27 ± 5.53%; *P* < 0.05), and 250 mg/kg (86.86 ± 3.00%; *P* < 0.05) compared with the MCAO ischemia group (Fig. 6A). Similarly, nitric oxide (NO) production was significantly decreased in the 62.5 mg/kg (71.00 ± 5.04%; *P* < 0.05), 125 mg/kg (71.79 ± 3.49%; *P* < 0.05), and 250 mg/kg (30.91 ± 3.24%; *P* < 0.05) treatment groups relative to the Ischemia group (Fig. 6B).

Furthermore, lipid peroxidation, assessed by thiobarbituric acid reactive substances (TBARS) levels, was significantly lower in mice treated with 62.5 mg/kg (87.36 ± 2.75%; *P* < 0.05), 125 mg/kg (87.88 ± 7.08%; *P* < 0.05), and 250 mg/kg (74.57 ± 6.41%; *P* < 0.05) of decanoic acid compared with the MCAO ischemia group (Fig. 6C). Collectively, these findings indicate that the neuroprotective effects of decanoic acid in ischemic stroke are mediated, at least in part, by its capacity to reduce oxidative stress, as evidenced by decreased ROS and NO production and diminished lipid peroxidation.

### Decanoic acid treatment modulated the activities of antioxidant enzymes

**Fig. 7:**
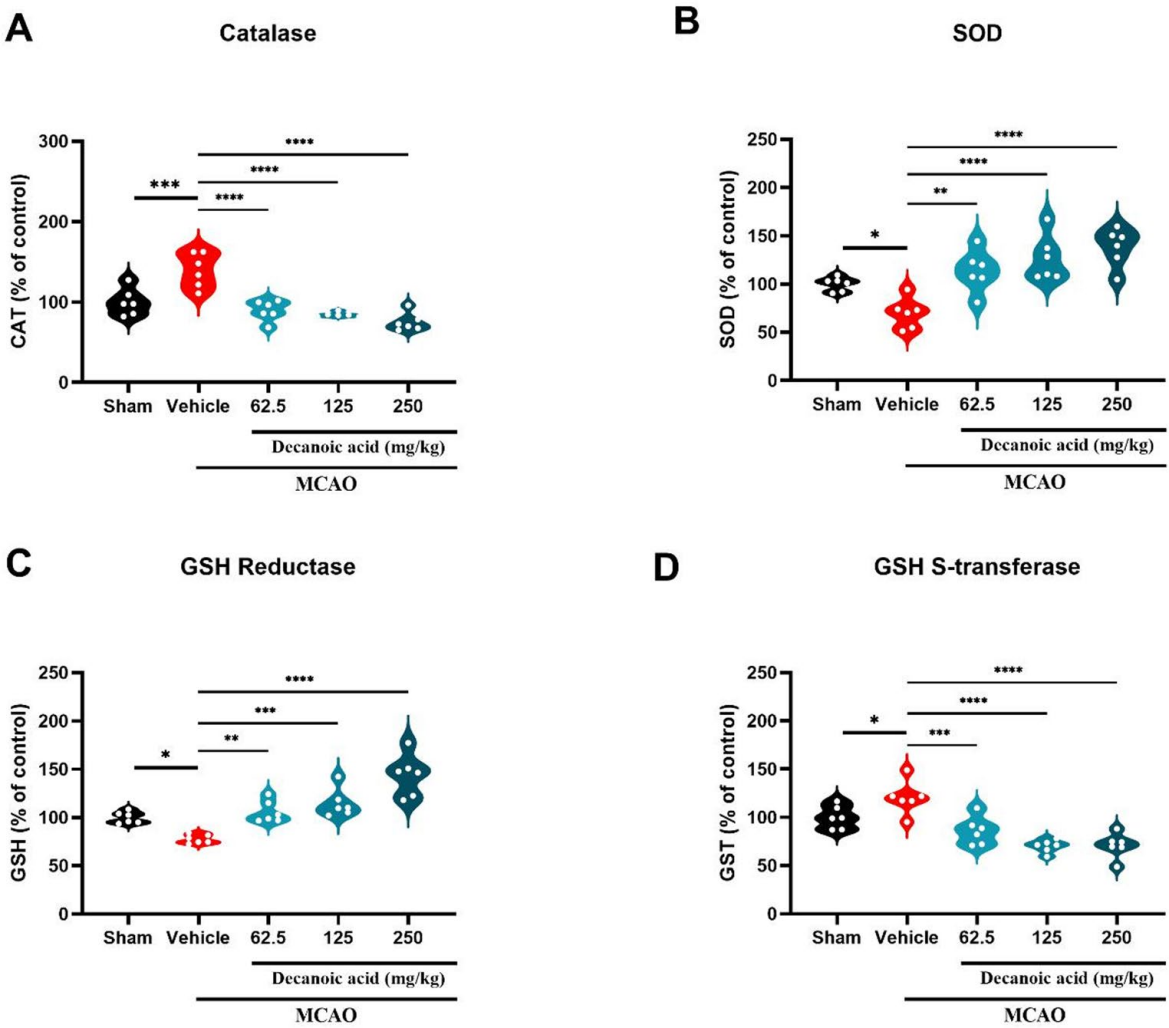
Decanoic acid modulates the activity of antioxidant enzymes

Concomitantly, the effects of decanoic acid on the antioxidant defense system were evaluated in a rodent model of cerebral ischemia. Glutathione reductase (GSH-Red) activity was significantly elevated in the groups with 62.5 mg/kg (105.93 ± 4.61%; *P* < 0.05), 125 mg/kg (114.74 ± 5.98%; *P* < 0.05), and 250 mg/kg (143.89 ± 3.49%; *P* < 0.05) compared with the MCAO ischemia group (Fig. 7A). Similarly, superoxide dismutase (SOD) activity was significantly increased in all treatment groups, 62.5 mg/kg (113.87 ± 8.64%; *P* < 0.05), 125 mg/kg (126.56 ± 9.55%; *P* < 0.05), and 250 mg/kg (138.60 ± 8.07%; *P* < 0.05), relative to the MCAO ischemia group (Fig. 7B).

In contrast, glutathione S-transferase (GST) activity was significantly reduced in mice treated with 62.5 mg/kg (85.95 ± 5.85%; *P* < 0.05), 125 mg/kg (70.18 ± 2.63%; *P* < 0.05), and 250 mg/kg (70.87 ± 5.23%; *P* < 0.05) compared with the MCAO ischemia group (Fig. 7C). Likewise, catalase (CAT) activity was markedly decreased in the 62.5 mg/kg (89.78 ± 5.08%; *P* < 0.05), 125 mg/kg (84.31 ± 1.12%; *P* < 0.05), and 250 mg/kg (75.06 ± 4.60%; *P* < 0.05) treatment groups (Fig. 7D). This reduction may reflect the normalization of oxidative status, potentially due to lower production of reactive oxygen species mediated by the enhanced activity of GSH-Red and SOD. Overall, these findings indicate that decanoic acid modulates the antioxidant defense system by increasing the activity of key antioxidant enzymes while decreasing the activity of enzymes typically upregulated under heightened oxidative stress conditions.

(**A**) Glutathione reductase (GSH-Red) activity, a key enzyme for redox homeostasis, significantly increased after treatment at all doses. (**B**) Superoxide dismutase (SOD) activity, responsible for converting superoxide anions into hydrogen peroxide, was markedly elevated. (**C**) Glutathione S-transferase (GST) activity, typically upregulated under oxidative stress, was reduced after treatment, suggesting lower oxidative burden. (**D**) Catalase (CAT) activity, which detoxifies hydrogen peroxide, was also reduced, likely reflecting diminished demand for antioxidant activity due to decreased ROS production. Data presented as mean ± SEM; one-way ANOVA followed by Dunnett’s post hoc test. *p* < 0.05; *n* = 6 per group.

### Decanoic acid treatment reduced pro-inflammatory markers in the ischemic cortex

**Fig. 8:**
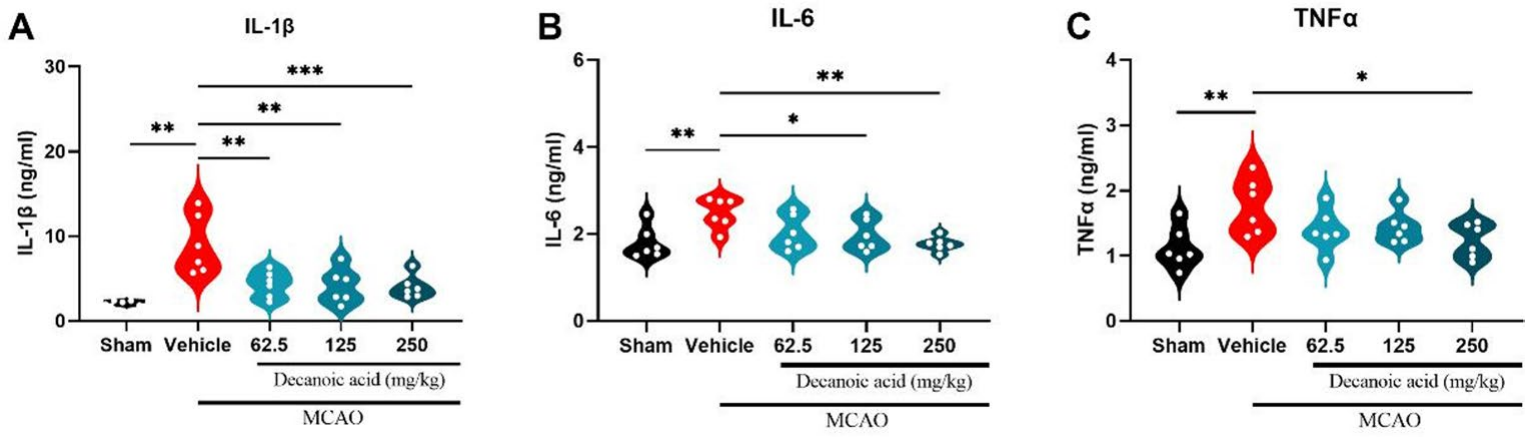
**Decanoic acid reduces pro-inflammatory cytokines in the ischemic cortex after cerebral ischemia.** Pro-inflammatory cytokines were quantified by ELISA in the supernatant of homogenized right ischemic cortex tissue collected on Day 8 after euthanasia. (**A**) Interleukin-1β (IL-1β) concentrations, significantly reduced in all treated groups. (**B**) Interleukin-6 (IL-6) levels also significantly decreased, particularly at doses of 125 and 250 mg/kg. (**C**) Tumor necrosis factor-α (TNF-α) concentrations, markedly reduced in the 250 mg/kg treatment group. Data are expressed as mean ± SEM and were analyzed using one-way ANOVA followed by Dunnett’s post hoc test. *p* < 0.05; *n* = 6 per group

Decanoic acid treatment promoted neuroprotection in the MCAO model, and metabolomic profiling revealed alterations in lipid and energy metabolism, including metabolites associated with inflammatory pathways. In light of these findings, pro-inflammatory cytokines were subsequently evaluated. For biochemical and inflammatory analyses, animals were euthanized on Day 8, and the right ischemic cortex was dissected, homogenized, and centrifuged; the resulting supernatant was used for cytokine quantification. After 7 days of treatment, interleukin-1β (IL-1β), interleukin-6 (IL-6), and tumor necrosis factor-α (TNF-α) levels were measured by ELISA in the supernatant of the homogenized right ischemic cortex (Fig. 8A–C).

Treatment with 62.5 mg/kg (4.31 ± 0.63; *P* < 0.05), 125 mg/kg (4.12 ± 0.84; *P* < 0.05), and 250 mg/kg (4.00 ± 0.55; *P* < 0.05) of decanoic acid significantly reduced IL-1β concentrations compared with the MCAO ischemia group (Fig. 8A). Similarly, IL-6 levels were significantly decreased in the 125 mg/kg (1.97 ± 0.14; *P* < 0.05) and 250 mg/kg (1.77 ± 0.06; *P* < 0.05) groups (Fig. 8B). TNFα concentrations followed the same pattern, with a significant reduction observed at the 250 mg/kg dose (1.23 ± 0.10; *P* < 0.05) compared to the MCAO ischemia group (Fig. 8C).

## Discussion

This study demonstrated that decanoic acid treatment produced significant improvements in both motor and cognitive performance in mice subjected to the middle cerebral artery occlusion (MCAO) model of permanent ischemia. In addition to reducing oxidative stress and neuroinflammation, decanoic acid markedly decreased infarct size, confirming its neuroprotective potential. A 7-day treatment regimen resulted in a consistent reduction in infarct area across all tested doses. The compound also effectively attenuated motor deficits, as evidenced by improved performance in limb-clasping, cylinder, and rotarod tests. These findings are in close agreement with those of Sharma et al. (2023), who reported that decanoic acid (120 mg/kg, oral) conferred significant neuroprotection in a transient MCAO model with 24 h of reperfusion, reducing infarct volume and improving motor coordination (Sharma et al. 2023a). In that study, the beneficial effects were associated with reduced oxidative stress and inflammation and upregulation of neurotrophic factors such as BDNF, TrkB, and NT-3. Together, these results strengthen the evidence that decanoyl supplementation mitigates ischemic brain injury through the modulation of oxidative and inflammatory pathways, supporting its potential as a neuroprotective agent in both transient and permanent ischemia.

Our results demonstrate that decanoic acid provides robust neuroprotection in the permanent MCAO model, markedly reducing infarct size and improving both motor and cognitive outcomes. These effects are accompanied by reduced oxidative stress and neuroinflammation, supporting the view that decanoyl supplementation mitigates ischemic injury through multiple converging mechanisms. The neuroprotective role of AMPA receptor antagonists in ischemic stroke has been widely documented. Nakashima et al. (2018) showed that perampanel, a selective AMPA receptor antagonist, significantly reduced infarct volume and improved motor performance in MCAO models (Nakajima et al. 2018). Similarly, other AMPA receptor inhibitors, including aniracetam, talampanel, YM872, EGIS-8332, GYKI53405, and β-caryophyllene, have demonstrated comparable efficacy in limiting ischemic brain injury (Chen et al. 2020; Erdő et al. 2006; Lo et al. 1997; Matucz et al. 2004; Sharma et al. 2023b; Takahashi et al. 2002). Collectively, these findings support the therapeutic potential of decanoic acid, given its non-competitive antagonism of AMPA receptors (Sharma et al. 2023a). Together, these findings strengthen the evidence that decanoic acid acts as a multifunctional neuroprotective agent, with benefits observed across both permanent and reperfusion-based ischemic paradigms.

Given that ischemia affected both the hippocampus and cortex, spatial and recognition memory were evaluated to assess the impact of decanoic acid on cognitive function. The open field test revealed no significant differences in locomotor activity among groups, confirming that the observed effects in the OLT and NORT were attributable to memory preservation rather than improvements in motor function. Decanoic acid treatment enhanced both spatial and recognition memory, suggesting its ability to mitigate ischemia-induced cognitive deficits. These findings agree with previous studies employing AMPA receptor inhibitors. For instance, Nakashima et al. (2018) demonstrated that perampanel reduced cortical neurodegeneration and improved spatial working memory in focal ischemia models (Nakajima et al. 2018). Similarly, Chen et al. (2020) reported that β-caryophyllene ameliorated cognitive impairment in mice following acute ischemic stroke (Chen et al. 2020). Sharma et al. (2023) further corroborated these results by showing that decanoic acid (120 mg/kg, oral) reduced infarct volume and upregulated BDNF and TrkB, key mediators of synaptic plasticity and cognitive recovery (Sharma et al. 2023a).While these findings collectively support a neuroprotective and pro-cognitive role for decanoic acid, further studies are warranted to evaluate synaptic remodeling and the expression of molecular markers associated with neuroplasticity.

The integrated chemometric, bioinformatic, and statistical analyses provide convergent evidence that treatment with decanoic acid (250 mg/kg) effectively mitigates ischemia-induced metabolic disturbances and promotes cerebral metabolic recovery. The multivariate chemometric analysis (PLS-DA) revealed distinct clustering among the Sham, MCAO, and decanoic acid-treated groups, with the treated animals positioned closer to the Sham condition—indicating partial restoration of metabolic homeostasis. VIP analysis identified key discriminant metabolites, including palmitoleamide, hexadecanal, and 1-methyl-1 H-pyrrole, which are linked to oxidative stress and lipid remodeling. This metabolic shift suggests that decanoic acid restores the balance between lipid degradation and synthesis pathways, reducing membrane peroxidation and enhancing mitochondrial lipid turnover after ischemic insult.

The bioinformatic pathway enrichment analysis corroborated these findings, revealing that ischemia profoundly disrupted fatty acid biosynthesis, β-oxidation of very long-chain fatty acids, and mitochondrial energy metabolism. Following treatment, partial reactivation of these pathways was observed, particularly in β-oxidation and lipid biosynthesis, indicating improved mitochondrial function and redox homeostasis. Moreover, the enrichment of pyruvate, amino sugar, and aspartate metabolism suggests enhanced metabolic flexibility, supporting the recovery of oxidative energy coupling and facilitating adaptation to post-ischemic metabolic stress. This reorganization of metabolic pathways highlights the capacity of decanoic acid to restore mitochondrial efficiency and sustain neuronal energy demand during recovery. Conventional statistical analysis reinforced these multivariate findings, showing that several metabolites altered by ischemia were partially normalized after treatment. Aldehydes derived from lipid peroxidation, such as octanal and tridecanal, and branched-chain amino acid catabolites, including 2-methyl-propanal and 3-methyl-butanal, exhibited recovery trends in decanoic acid-treated animals, consistent with attenuation of oxidative stress and restoration of mitochondrial metabolism. Importantly, the reestablishment of cerebral n-decanoic acid levels confirms its effective uptake and metabolic incorporation, validating its central action in modulating lipid homeostasis and β-oxidation. Taken together, the integration of chemometric, bioinformatic, and statistical analyses demonstrates that decanoic acid exerts a coordinated regulatory effect on cerebral metabolism, reestablishing lipid integrity, redox balance, and mitochondrial energy dynamics.

Given the strong association between these metabolic pathways and redox balance, we next sought to evaluate oxidative stress markers to further elucidate the mechanisms underlying the neuroprotective effects of decanoic acid. In line with the known pathophysiological cascade of ischemic injury, cerebral ischemia induced excessive ROS production, a consequence of mitochondrial dysfunction driven by oxygen and energy substrate deprivation, collapse of the electron transport chain, and increased free radical leakage (Kim et al. 2015; Wu et al. 2020). As expected, the MCAO model exhibited elevated levels of ROS, NO, and TBARS, confirming enhanced oxidative stress. Remarkably, 7 days of decanoic acid treatment significantly reduced these oxidative markers, indicating the activation of endogenous antioxidant defense mechanisms. These findings are consistent with previous reports describing the antioxidant properties of decanoic acid in both in vitro (Mett and Müller 2021) and in vivo (Sharma et al. 2023a) models.

Further analysis of endogenous antioxidant enzyme activities revealed increased superoxide dismutase (SOD) and glutathione reductase (GSH-Red) activity in the decanoic acid–treated groups, indicating an enhanced first-line antioxidant defense. SOD catalyzes the dismutation of superoxide anion (O₂⁻) into hydrogen peroxide (H₂O₂) and molecular oxygen, whereas GSH-Red facilitates the regeneration of reduced glutathione (GSH), a critical cofactor for detoxifying reactive species (Cherubini et al. 2005), (Zimmermann et al. 2004). In contrast, glutathione S-transferase (GST) and catalase (CAT) activities were significantly reduced following decanoic acid treatment. GST enzymes conjugate GSH with lipid peroxidation products, enabling their detoxification (Higashi et al. 2021), while CAT catalyzes the breakdown of H₂O₂ into water and oxygen (Pinto et al. 2012). The observed reduction in GST and CAT activity suggests a lower demand for detoxification, consistent with the decreased lipid peroxidation levels observed in treated groups. Although increased SOD activity leads to higher H₂O₂ production, the combination of enhanced antioxidant capacity and reduced upstream ROS generation results in an overall decrease in oxidative burden, diminishing the requirement for elevated CAT activity.

Neuroinflammation plays a pivotal role in the progression of ischemic injury, with microglial and astrocytic activation contributing to neuronal degeneration through the release of pro-inflammatory cytokines (Loddick and Rothwell 1996; McCoy and Tansey 2008). The excessive secretion of IL-1β, IL-6, and TNFα exacerbates neuronal damage following stroke (Allan et al. 2005; Loddick and Rothwell 1996; McCoy and Tansey 2008; Mulcahy et al. 2003). Notably, decanoic acid treatment reversed the ischemia-induced elevation of IL-1β, IL-6, and TNFα levels, effectively neutralizing the pro-inflammatory surge triggered by the occlusion. These results are consistent with previous evidence showing that medium-chain fatty acids modulate neuroinflammatory pathways, attenuating cytokine release and glial activation (Dupuis et al. 2015; Jeong et al. 2011; Ruskin et al. 2009). Moreover, Sharma et al. (2023) reported similar findings in a transient MCAO model, in which decanoic acid administration markedly decreased pro-inflammatory cytokines (TNF-α, IL-1β, and IL-6) and increased IL-10 expression, reinforcing the view that decanoyl supplementation counteracts ischemia-induced neuroinflammation by promoting a shift toward an anti-inflammatory profile (Sharma et al. 2023a). Collectively, these findings suggest that decanoic acid may exert a coordinated regulatory effect, potentially contributing to the modulation of cerebral metabolic homeostasis and attenuation of the pro-inflammatory response triggered by ischemia.

## Conclusion

The present study demonstrates that a 7-day oral treatment with decanoic acid effectively reduces infarct size and mitigates motor and cognitive deficits in mice subjected to the MCAO model of cerebral ischemia. Among the doses assessed, 250 mg/kg produced the most pronounced effects. Mechanistic analyses revealed that the neuroprotective actions of decanoic acid involve attenuation of oxidative stress evidenced by reduced reactive oxygen species (ROS) production enhancement of antioxidant enzyme activity, and modulation of the inflammatory response by counteracting the pathological increase in pro-inflammatory cytokine expression, thereby promoting a shift toward an anti-inflammatory profile.

These findings provide robust evidence supporting the therapeutic potential of decanoic acid in limiting ischemic brain injury and promoting both functional and cognitive recovery. Furthermore, they expand the understanding of medium-chain fatty acids as neuroprotective agents. While the results position decanoic acid as a promising candidate for stroke therapy, further research is warranted to elucidate its precise molecular targets, evaluate long-term efficacy, and assess its translational potential in clinical settings. Such insights may contribute to the development of novel therapeutic strategies aimed at enhancing neuroprotection and recovery in patients with ischemic stroke. 

## Supplementary Information

Supplementary Material 1.

## Data Availability

The datasets generated during the current study are available from the corresponding author on request.
